# Rhizosphere bacterial communities of dominant steppe plants shift in response to a gradient of simulated nitrogen deposition

**DOI:** 10.3389/fmicb.2015.00789

**Published:** 2015-08-12

**Authors:** An Yang, Nana Liu, Qiuying Tian, Wenming Bai, Mark Williams, Qibing Wang, Linghao Li, Wen-Hao Zhang

**Affiliations:** ^1^State Key Laboratory of Vegetation and Environmental Change, Institute of Botany, Chinese Academy of SciencesBeijing, China; ^2^University of Chinese Academy of SciencesBeijing, China; ^3^Department of Horticulture, Virginia Polytechnic Institute and State UniversityBlacksburg, VA, USA; ^4^Research Network of Global Change Biology, Beijing Institutes of Life Science, Chinese Academy of SciencesBeijing, China

**Keywords:** nitrogen deposition, microbial diversity, rhizosphere, Illumina Miseq, temperate steppe, *Artemisia frigida*, *Stipa kerlovii*

## Abstract

We evaluated effects of 9-year simulated nitrogen (N) deposition on microbial composition and diversity in the rhizosphere of two dominant temperate grassland species: grass *Stipa krylovii* and forb *Artemisia frigida.* Microbiomes in *S. krylovii* and *A. frigida* rhizosphere differed, but changed consistently along the N gradient. These changes were correlated to N-induced shifts to plant community. Hence, as plant biomass changed, so did bacterial rhizosphere communities, a result consistent with the role that N fertilizer has been shown to play in altering plant-microbial mutualisms. A total of 23 bacterial phyla were detected in the two rhizospheric soils by pyrosequencing, with *Proteobacteria, Acidobacteria*, and *Bacteroidetes* dominating the sequences of all samples. *Bacterioidetes* and *Proteobacteria* tended to increase, while *Acidobacteria* declined with increase in N addition rates. *TM7* increased >5-fold in the high N addition rates, especially in *S. krylovii* rhizosphere. Nitrogen addition also decreased diversity of OTUs (operational taxonomic units), Shannon and Chao1 indices of rhizospheric microbes regardless of plant species. These results suggest that there were both similar but also specific changes in microbial communities of temperate steppes due to N deposition. These findings would contribute to our mechanistic understanding of impacts of N deposition on grassland ecosystem by linking changes in plant traits to their rhizospheric microbes-mediated processes.

## Introduction

Nitrogen (N), an essential mineral nutrient to plant growth, is one of the most limiting factors in many terrestrial ecosystems (Vitousek et al., 2002; Scheible et al., 2004; Asakawa and Kimura, 2008). However, elevated levels of N deposition are changing N inputs and impacting many ecological processes (Sala et al., 2000; Gilliam, 2006). Many ecosystems have developed under tight nutrient cycling and low amounts of available nutrients. In these systems, elaborate plant-microbial mutualisms have developed and are the foundation of ecosystem function (Reynolds et al., 2003). Nitrogen deposition can disrupt these plant-microbial interactions, which may feedback to alter microbial communities and ecosystem function (Sala et al., 2000; Gilliam, 2006; Martinelli et al., 2006).

Human industrial activities have led to a doubling, on average, of N deposition into terrestrial ecosystems and these inputs are expected to increase in the future (Galloway et al., 2004, 2008; Bodirsky et al., 2014). In China, N deposition has significantly increased over the last three decades, and in some regions are 5X greater than previous decades (Liu et al., 2013). Consequently, the impacts of N input due to deposition of atmospheric N on terrestrial ecosystems warrant further study (Galloway et al., 2004, 2008).

Grassland ecosystems are highly sensitive to N deposition, where long-term N addition has been shown to significantly reduce plant species richness (Stevens et al., 2004; Clark and Tilman, 2008). The semi-arid grasslands in northern China, which are a part of the Eurasian steppe, are exposed to enhanced N deposition rates (Zhang et al., 2008; Liu et al., 2013), and experienced reductions in plant species richness (Bai et al., 2010; Song et al., 2011; Fang et al., 2012; Tian et al., 2015). Several mechanisms have been proposed to explain the decline in species richness by N deposition (Suding et al., 2005; Harpole and Tilman, 2007; Bobbink et al., 2010), and among them are changes in soil microbial activity and biodiversity (Chen et al., 2014; Dean et al., 2014). Nitrogen deposition to a variety of terrestrial ecosystems has also been shown to profoundly affect soil microbial communities (Ramirez et al., 2010, 2012; He et al., 2013; Liu et al., 2014b; Zhang et al., 2014), however, these studies have not emphasized the involvement of root-zone and rhizosphere microbes; rather they have mainly focused on microbes in soils generally. The rhizosphere niche is an important interface for plant-microbes interactions and key to the success of both plants and microbes (Bakker et al., 2013). Microbe communities in the rhizosphere soils differ from those in bulk soils (Berg and Smalla, 2009), thus deserving specific attention for understanding ecosystem responses to N deposition. It is conceivable that microbial communities in the rhizosphere of different plant species may respond more strongly and perhaps, differently, to N deposition. Recently, Dean et al. (2014) found that N deposition affected host-associated plant root-associated fungi, however, there was no broader description of fungal or rhizosphere bacterial communities.

In the present study, we found that plant community shifted from co-dominance by a monocot grass, *Stipa krylovii*, and a dicot forb, *Artemisia frigida*, to exclusive dominance by a monocot grass in an Inner Mongolia steppe after 9-year of N addition. To test whether the microbial communities colonizing *S. krylovii* and *A. frigida* rhizosphere niche respond, and respond in similar or different ways to N addition, we used the high-throughput Illumina Miseq sequencing platform to characterize the rhizosphere microbial communities of the two dominant plant species under varying simulated N deposition rates. The following question was specifically addressed: Whether the rhizosphere microbial communities would be associated with plant host and its response to N deposition rate?

## Materials and methods

### Study site

The experiment was conducted in Duolun county (42°02′N, 116°17′E, 1324 m a.s.l.), Inner Mongolia, China. The area is located in a semiarid temperate steppe where the mean annual temperature is 2.1°C and long-term annual precipitation is 382.2 mm (Yang et al., 2011; Fang et al., 2012). The soil in the area is chestnut (Chinese classification) and Haplic Calcisols (FAO classification). The soil bulk density is 1.31 g cm^−3^ and pH is about 6.84 (Fang et al., 2012). The dominant species in this typical temperature steppe are *A. frigida, S. krylovii, Cleistogenes squarrosa, Allium bidentatum, Potentilla acaulis, Leymus chinensiss, Salsola collina, Carex korshinskyi, Melilotoides ruthenica*, and *Agropyron cristatum* (Niu et al., 2008; Fang et al., 2012).

### Experimental design

In the experimental area, 64 plots of 15 × 10 m separated by 4-m-wide buffer strips were established in an 8 × 8 Latin square experimental design. Nitrogen was added as urea (N, 46%) at the midpoint of the growing season (July) every year since 2003. There were eight levels of N fertilization including a control, 0 (N0), 1 (N1), 2 (N2), 4 (N4), 8 (N8), 16 (N16), 32 (N32), and 64 (N64) g N m^−2^ year^−1^ with ambient N deposition of 1.6 g N m^−2^ year^−1^ (Zhang et al., 2008). In the present study, the rhizosphere soil samples were collected from 32 plots with four levels of N fertilization, ambient (N0), 2 (N2), 8 (N8), and 16 (N16) g N m^−2^ year^−1^. The rhizospheric soils from at least two individual plant roots of the two dominant species (*S. krylovii, A. frigida*) in each plot were sampled in August 2012 by collecting soils that were adhered to roots after vigorously shaking roots removed from field by a spade as described by Smalla et al. (2001). The rhizospheric soils were sampled from the eight plots under the four N addition levels, and the final three soil samples used for sequencing were obtained by randomly mixing the samples from 3, 3, and 2 plots, respectively. These ensure that the rhizospheric soil samples covered the eight replicates for N addition. Aboveground biomass in one quadrat (1 × 1 m) at each plot was clipped and determined since August 2004 as described by Fang et al. (2012).

### Sequencing and data analysis

The total genomic DNA was extracted from 0.5 g rhizosphere soils using the SoilGen DNA Kit (CWbiotech Corporation, China) according to the manufacturer's instructions. PCR amplifications were conducted with the 515f/806r (GTGCCAGCMGCCGCGGTAA/GGA CTACHVGGGTWTCTAAT) primer set that amplified the V4 region of the 16S rRNA gene (Peiffer et al., 2013). The primer set was selected as it exhibits few biases and should yield accurate taxonomic information. The reverse primer contained a 6-bp error-correcting barcode unique to each sample. The PCR reaction was carried out in 30 μL reactions with 15 μL of Phusion® High-Fidelity PCR Master Mix (New England Biolabs); 0.2 μM of forward and reverse primers, and about 10 ng template DNA. Thermal cycling was consisted of initial denaturation at 98°C for 1 min, then 30 cycles of denaturation at 98°C for 10 s, annealing at 50°C for 30 s, elongation at 72°C for 60 s and finally 72°C for 5 min. PCR products were mixed in equal density ratios. Then, mixture PCR products were purified with GeneJET Gel Extraction Kit (Thermo Scientific). Sequencing libraries were generated using NEB Next® Ultra™ DNA Library Prep Kit for Illumina (NEB, USA) following manufacturer's recommendations and index codes were added. The library quality was assessed on the Qubit @ 2.0 Fluorometer (Thermo Scientific) and Agilent Bioanalyzer 2100 system. At last, the library was sequenced on an Illumina MiSeq platform and 300 bp paired-end reads were generated.

Pairs of reads from the original DNA fragments were merged by using FLASH-a very fast and accurate software tool which was designed to merge pairs of reads when the original DNA fragments were shorter than twice the length of reads (Magoc and Salzberg, 2011). Sequencing reads were assigned to each sample according to the unique barcode of each sample. Sequences were analyzed with the QIIME software package (Quantitative Insights Into Microbial Ecology) and UPARSE pipeline (Caporaso et al., 2010; Edgar, 2013). First, the reads were filtered by QIIME quality filters. Default settings for Illumina processing in QIIME were used. Then we used UPARSE pipeline to pick operational taxonomic units (OTUs) by making OTU table. After removal of chimera, sequences were assigned to OTUs at 97% similarity. We picked a representative sequence for each OTU and used the version 2.2 RDP classifier to assign taxonomic data to each representative sequence with default 0.8 as confidence threshold (Wang et al., 2007). Singleton OTUs that appeared in only one sample were removed because they could be potential sequencing errors. In order to compute alpha diversity, we rarified the OTU table and calculated three metrics: Chao1 metric estimated the species richness, the observed species metric was simply the count of unique OTUs found in the sample, and Shannon index. Rarefaction curves were generated based on these three metrics. QIIME calculated unweighted unifrac, which was used to do Principal Coordinate Analysis (PCA).

### Sequence accession number

The data were deposited in the National Center for Biotechnology Information Sequence Reads Archive with accession number SRS977347.

### Statistical analysis

Considering that the N addition and plant host in the same plot may not be completely independent, we used the linear mixed models to analyze the effects of N addition on the rhizoshpere microbes at four levels with N treatment as the fixed effect and plant host as random effects. For each species, we conducted separate ANOVAs (Dunnett's test) to determine the difference in the aboveground biomass (AGB), microbial diversity and relative abundance of phyla between N0 and different levels of N addition (SPSS 16.0). It was regarded as significant differences when *P*-value was less than 0.05. Square root transformation of the OTUs data and arcsine square root transformation of relative abundance of phyla were done before proceeding with ANOVA and *post-hoc* test. The assumptions of normality and homogeneity of variance were checked prior to conducting the statistical tests.

## Results

### Nitrogen addition reduced and enhanced aboveground biomass (AGB) of forbs and grasses

**Figure 1:**
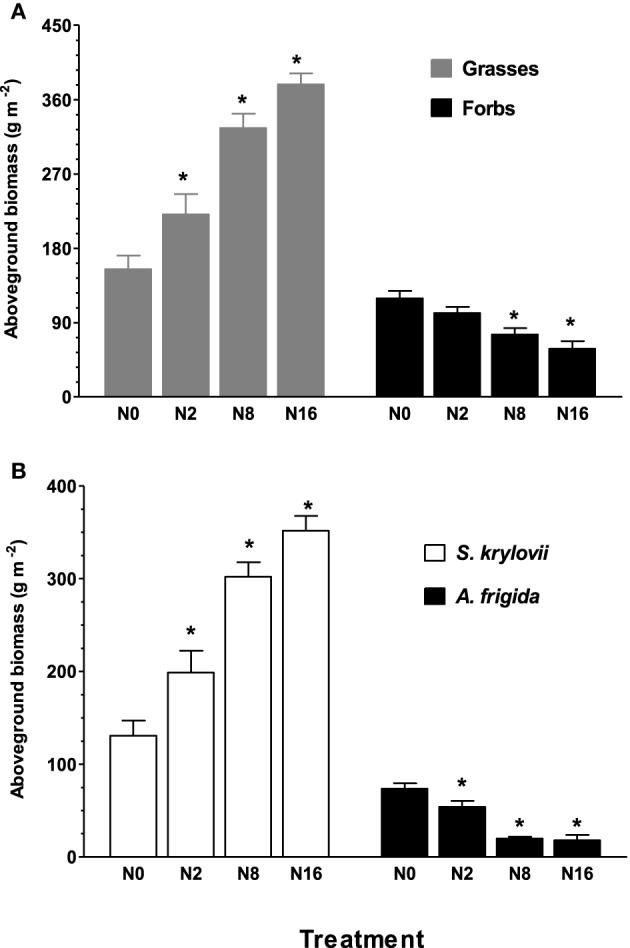
**Effect of N addition on aboveground biomass of grasses and forbs (A), and *S. krylovii* and *A. frigida* (B)**. Aboveground biomass was determined in quadrats (1 × 1 m). N0, N2, N8, and N16 represent N addition rate of 0, 2, 8, 16 g ha^−1^ yr^−1^. Data are means ± s.e. (*n* = 8). Asterisks on the top of columns indicate significant difference at *P* < 0.05 between N0 and different rates of N addition for each species.

Nitrogen addition for 9 years had contrasting effects on AGB of grasses and forbs, such that AGB of grasses and forbs was significantly (*P* < 0.05) increased and reduced by N addition, respectively (Figure 1A). The steppe community was co-dominated by grass *S. krylovii* and forb *A. frigida* in the control plot without N addition. The AGB of *S. krylovii* was enhanced by the N addition, while the same treatment led to a decrease in AGB of *A. frigida* (*P* < 0.05) (Figure 1B).

### Sequencing and analysis of rhizospheric microbial diversity

**Figure 2:**
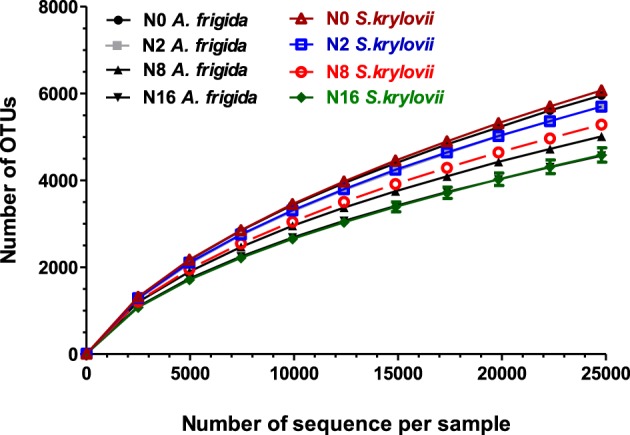
**Rarefaction curves of all samples were generated for microbial OTUs which contained unique sequences and were defined at 97% sequence similarities**.

We obtained a total of 1,931,731 clean reads after filtered by QIIME quality filters with default settings (Table A1). One sample from N0 plots was later excluded from analysis because of the low quality of the sequence reads. Rarefaction analysis was performed on each soil sample and none of the rarefaction curves reached the plateau phase, suggesting that soils were not sampled to saturation (Figure 2).

**Figure 3:**
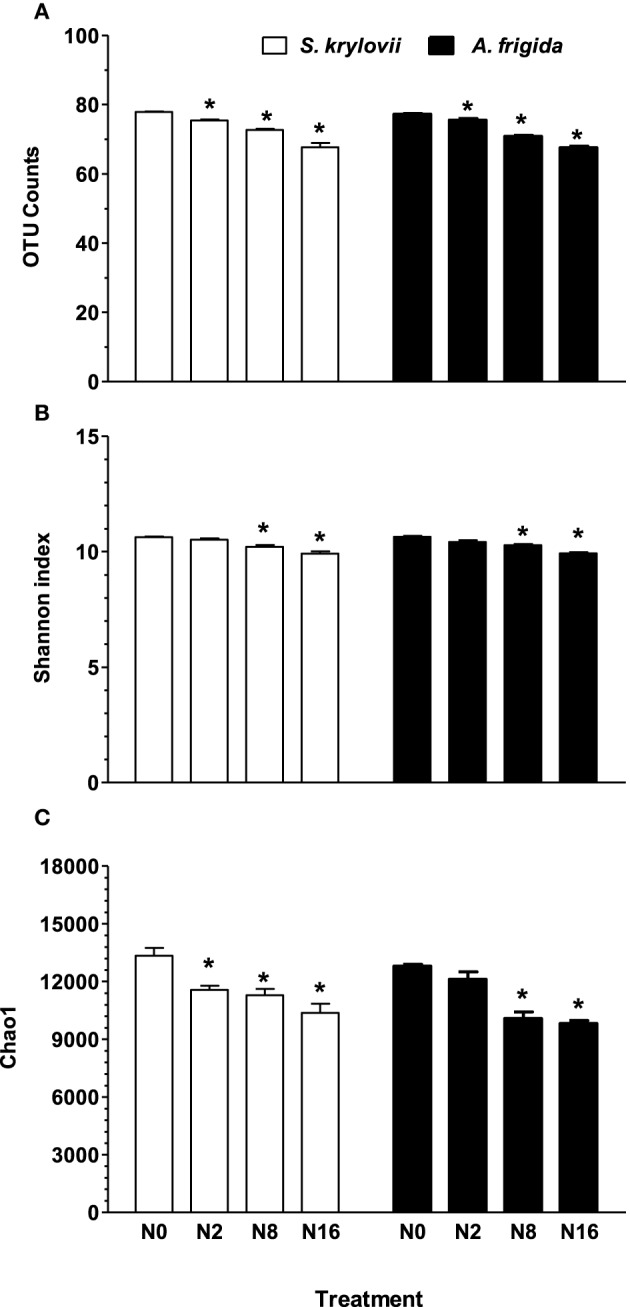
**Estimated number of observed (A) OTU counts, (B) Shannon index, (C) Chao1 index of *S. krylovii* and *A. frigida* rhizosphere microbiome across all the N-supplied plots**. Data are means ± s.e. (*n* = 3). Asterisks on the top of columns indicate significant difference at *P* < 0.05 between N0 and different rates of N addition for each species.

Linear mixed model analysis indicated that OTU number, Chao1, and Shannon indices of rhizosphere microbes of both *S. krylovii* and *A. frigida* were significantly affected by the N addition (Figure 3 and Table 1). In control plot without N addition, the number of OTUs in the rhizosphere of *S. krylovii* and *A. frigida* was 6036 and 5957, respectively (Figure 3A). Nitrogen addition led to similar effects on microbial diversity in the rhizosphere of the two species. As N addition rate increased, the OTU number in both the *S. krylovii* and *A. frigida* rhizospheric niche decreased (Figure 3A). For example, the OTU number in the *S. krylovii* rhizosphere was decreased from 6036 to 5698, 5282, and 4583 in response to N addition rate of 2, 8, and 16 g N m^−2^ yr^−1^. Similarly, the OTU number in the *A. frigida* rhizosphere niche decreased from 5957 to 5700, 5010, and 4567 in response to the same N addition rates (Figure 3A). In addition to the number of OTUs, changes in microbial diversity using the Shannon index and the total species richness estimated by the Chao1 index in the two rhizospheric soil samples were also compared among plots treated with different rates of N addition. Shannon and Chao1 indices in the two rhizospheric soils also showed similar trends with increases in N addition rates, such that N addition reduced Shannon and Chao1 indices in the rhizosphere of *S. krylovii* and *A. frigida* (Figures 3B,C).

**Table 1:** **F-value and *P*-value of analysis of variance for the effects of nitrogen addition, species and their interaction (nitrogen × species) on OTU, Shannon, Chao1 indices and the relative abundance of the phyla**.

**Fixed factors**		**Nitrogen**	**Species**	**Nitrogen × Species**
d.f.		3	1	3
OTU	*F*	252.74	7.99	3.18
	*P*	< 0.0001	0.0143	0.06
Shannon	*F*	75.03	0	1.22
	*P*	< 0.0001	0.9624	0.3424
Chao1	*F*	49.09	5.20	4.41
	*P*	< 0.0001	0.0400	0.0239
*Acidobacteria*	*F*	146.93	13.67	11.94
	*P*	< 0.0001	0.0027	0.0005
*Proteobacteria*	*F*	19.53	9.94	1.04
	*P*	< 0.0001	0.0076	0.4057
*Bacteroidetes*	*F*	30.34	0.09	9.87
	*P*	< 0.0001	0.7733	0.0012
*Crenarchaeota*	*F*	72.80	0.01	11.96
	*P*	< 0.0001	0.9247	0.0005
*Verrucomicrobia*	*F*	4.03	10.84	0.37
	*P*	0.0314	0.0058	0.7754
*Planctomycetes*	*F*	6.24	1.42	3.27
	*P*	0.0074	0.2555	0.0559
*Actinobacteria*	*F*	4.71	5.65	4.62
	*P*	0.0194	0.0335	0.0207
*Cyanobacteria*	*F*	0.62	0	0.41
	*P*	0.6168	0.9601	0.7504
*Gemmatimonadetes*	*F*	12.42	7.81	5.34
	*P*	0.0004	0.0152	0.0129
*Chloroflexi*	*F*	16.22	15.54	6.55
	*P*	0.0001	0.0017	0.0062
*Firmicutes*	*F*	39.78	21.00	0.28
	*P*	< 0.0001	0.0005	0.8384
*WYO*	*F*	1.75	24.18	11.48
	*P*	0.2057	0.0003	0.0006
*TM7*	*F*	195.38	5.60	7.16
	*P*	< 0.0001	0.0341	0.0044

**Figure 4:**
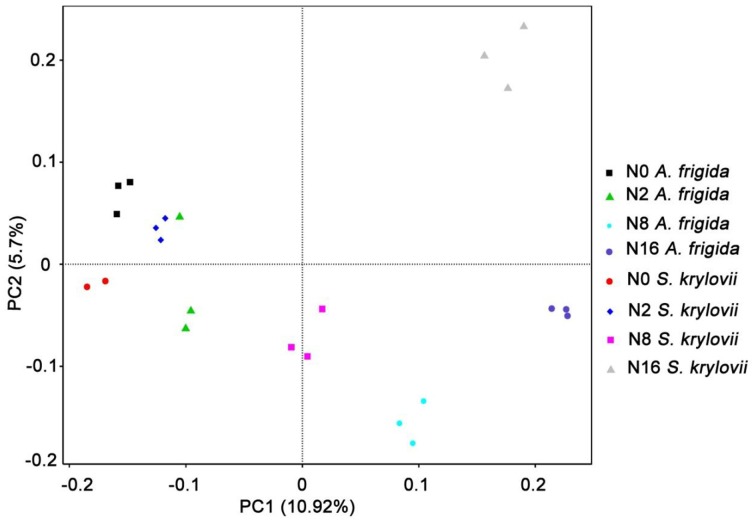
**Principle component analysis (PCA) of microbial communities based on OTUs for all samples from *S. krylovii* and *A. frigida* rhizosphere**. The first two components were 10.68% and 5.46%, respectively.

Principal component analysis was used to detect variation in the community composition. As shown in Figure 4, the two principal components accounted for 16.62% of the total microbial community variations among the individual samples. The two-dimensional figure showed that the microbial community compositions in both *S. krylovii* and *A. frigida* rhizosphere niche with different N addition rates were distributed separately among each other, exhibiting differences in the microbial community structure. These results suggest that the *S. krylovii* and *A. frigida* rhizosphere microbiome had different composition and that the composition changed along the N-gradient.

### Effect of N addition on relative abundance of rhizospheric microbe

**Figure 5:**
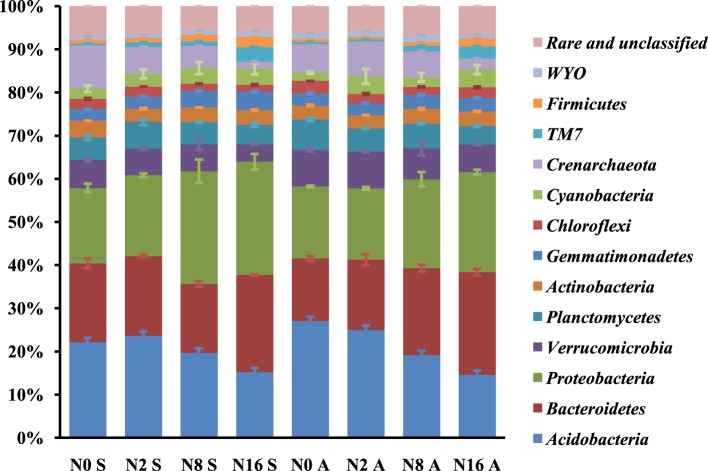
**The relative abundance of phyla of *S. krylovii* and *A. frigida* rhizosphere microbiome across all the N-supplied plots (% of total sequence)**.

Analysis of the taxonomic groups detected in the soil samples showed that there were a total of 23 phyla in the rhizosphere of both *S. krylovii* and *A. frigida*. The most dominant phyla across all samples were *Proteobacteria, Acidobacteria*, and *Bacteroidetes*, accounting for about 60% of the bacterial sequences (Figure 5). In addition, *Verrucomicrobia, Crenarchaeota, Planctomycetes, Actinobacteria, Cyanobateria, Gemmatimonadetes, Chloroflexi, TM7, Firmicutes*, and *WYO* were detected in all the samples with low abundance, while the unclassified and rare phyla accounted for about 6.3% in the samples. In the control plots without N addition, the relative abundance of *Bacteroidetes* and *Crenarchaeota* in the rhizosphere of *S. krylovii* was higher than that in the rhizosphere of *A. frigida* (*P* < 0.05). In contrast, the relative abundance of *Acidobacteria, Verrucomicrobia, Chloroflexi*, and *WYO* in the rhizosphere of *S. krylovii* was lower than that in the rhizosphere of *A. frigida* (*P* < 0.05) (Figure 5). These results suggest that difference existed between the microbial communities in the rhizosphere of both *S. krylovii* and *A. frigida* in the control plot.

**Figure 6:**
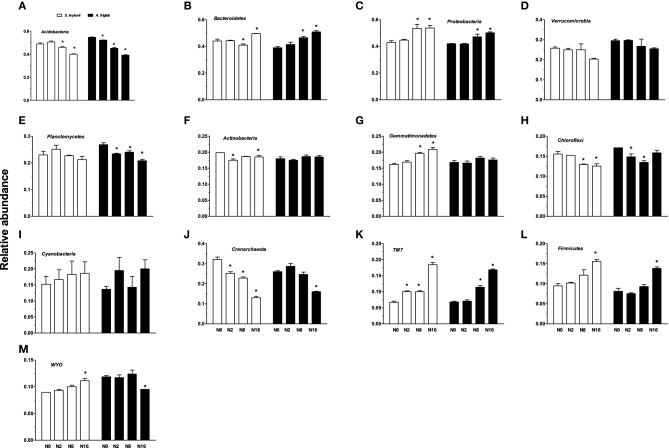
**Effect of N addition on the relative abundance of phyla A, of *S. krylovii* and *A. frigida* rhizosphere microbiome across all the N-supplied plots**. **(A)** Acidobacteria, **(B)** Bacteroidetes, **(C)** Proteobacteria, **(D)** Verrucomicrobia, **(E)** Planctomycetes, **(F)** Actinobacteria, **(G)** Gemmatimonadetes, **(H)** Chloroflexi, **(I)** Cyanobacteria, **(J)** Crenarchaeota, **(K)** TM7, **(L)** Firmicutes, **(M)** WYO. Data are means ± s.e. (*n* = 3). Asterisks on the top of columns indicate significant difference at *P* < 0.05 between N0 and different rates of N addition for each species.

Nitrogen addition had significant impacts on the relative abundance of the phyla (Figures 5, 6). With the increases in N addition rates, the relative abundance of *Bacteroidetes, Proteobacteria, Gemmatimonadetes, TM7, Firmicutes*, and *WYO* was enhanced in the *S. krylovii* rhizosphere (Figures 6B,C,G,K–M). A decrease in the relative abundance of *Acidobacteria, Actinobacteria, Chloroflexi*, and *Crenarchaeota* in the *S. krylovii* rhizosphere niche was detected (Figures 6A,F,H,J), while the relative abundance of *Verrucomicrobia, Planctomycetes*, and *Cyanobacteria* in the *S. krylovii* rhizosphere niche remained relatively unchanged (Figures 6D,E,I). With the increases in N addition rates, the relative abundance of *Bacteroidetes, Proteobacteria, TM7*, and *Firmicutes* in the *A. frigida* rhizosphere niche was increased (Figures 6B,C,K,L). Nitrogen addition had little effects on the relative abundance of *Verrucomicrobia, Actinobacteria, Gemmatimonadetes*, and *Cyanobacteria* in the *A. frigida* rhizosphere niche (Figures 6D,F,G,I). By contrast, the relative abundance of *Acidobacteria, Planctomycetes, Chloroflexi, Crenarchaeota*, and *WYO* in the *A. frigida* rhizosphere niche was reduced by N addition (Figures 6A,E,H,J,M).

## Discussion

In this study, we found that 9-year N addition shifted plant community structure from co-dominance by a monocot grass, *S. krylovii*, and a dicot forb, *A. frigida*, to exclusive dominance by a monocot grass in an Inner Mongolia steppe (Figure 1). These results concurred with other studies which showed that N enrichment favors more for grass growth than for forb growth (Song et al., 2011; Fang et al., 2012; Zhang et al., 2014). The differential growth responses of the two plants to N addition prompted us to test whether N addition may also have different impacts on the microbial communities in the rhizosphere of the two species. We thus characterized microbial communities in the rhizosphere of the two dominant species, *S. krylovii* and *A. frigida*, under varying rates of N addition by high-throughput sequencing technique. Our results showed that the relative abundance of most microbial phyla in the rhizosphere of *S. krylovii* differed from that of *A. frigida* under control, ambient N conditions, suggesting that the microbiomes in the rhizosphere of the two species differ intrinsically. Bakker et al. (2013) have suggested that root exudates can explain the differences in the rhizospheric microbiomes of various plant species. In this context, our previous studies showed that *S. krylovii* and *A. frigida* differed in their rhizospheric processes, such that *S. krylovii* roots can exude greater amount of organic anions (malate and citrate) than *A. frigida* roots under ambient N conditions (Liu et al., 2014a). In addition, *Acidobacteria, Bacteroidetes*, and *Proteobacteria* were dominant microbial species in the two rhizosphere microbiomes, accounting for about 60% of the total microbiomes, implying that the three phyla may play important roles in the rhizosphere of the two species.

Several studies have reported that elevated N deposition profoundly impacts the soil microbial communities across different terrestrial ecosystems (Ramirez et al., 2010, 2012; He et al., 2013; Zhang et al., 2014). The microbes in the bulk soils have often been used to evaluate the effect of N addition on composition and biodiversity of bacterial community. However, in contrast to our studies, few studies have specifically investigated the effect of N addition on rhizospheric microbial communities. In a recent study, Zhu et al. (2015) reported that short-term N addition has minimal influence on rhizosphere effect of smooth crabgrass and bermudagrass by T-RFLP and analysis of enzyme activities. In the present study, we examined the rhizosphere microbiomes of *S. krylovii* and *A. frigida* across different N addition rates. We found that the OTU number, Shannon and Chao1 indices in the rhizospheric soils of the two dominant species showed a similar trend in response to N addition. Nitrogen addition reduced Shannon index in the rhizosphere of *S. krylovii* and *A. frigida*, indicating that the rhizospheric microbiomes of the two species became less diverse with increases in N-addition rates. In a similar study, Zhang et al. (2014) reported that long-term N addition had no effects on the overall OTU number of bulk soils in the Inner Mongolia steppe. Given the high heterogeneity of soils and co-existence of multiple plant species in a natural ecosystem, results obtained from monitoring microbes in the bulk soils may not truly reflect the changes in rhizospheric microbial communities. Roots are high in C, so the microbial community closer to the root may be copiotrophic relative to those in bulk soils (Nguyen, 2003; Badri et al., 2009; Gottel et al., 2011). We also found that the relative abundance of most phyla was altered in response to the long-term N addition. The relative abundance of all phyla except *Verrucomicrobia, Planctomycetes*, and *Cyanobacteria* in the rhizospheric microbiomes of *S. krylovii* was altered in response to the N addition. In the rhizospheric microbiomes of *A. frigida*, we found that the relative abundance of *Verrucomicrobia, Actinobacteria, Gemmatimonadetes*, and *Cyanobacteria* remained relatively unchanged by the same N addition. Furthermore, our results showed that the direction of the response of individual phyla to N addition was dependent upon host identity. These findings are in contrast to those previously reported results, where no comparable increases and decreases in response to N addition were observed in the same bacterial phylum of grasslands (Ramirez et al., 2010, 2012; He et al., 2013; Liu et al., 2014b). The differences in soil sampling methods (rhizospheric soils vs. bulk soils) between our studies and others may partly account for the different results. It is more likely that microbes in the rhizosphere simply behave differently from those in bulk soils.

It has been established that interaction can occur between plant communities and soil microbial communities (Zak et al., 2003; Dean et al., 2014). Many studies have attempted to link the above-ground plant diversity and productivity to below-ground bacterial diversities by characterizing soil bacterial communities (Zak et al., 2003; Dean et al., 2014). Rooney et al. (2006) showed that in agricultural grasslands the response of plants and bacterial communities to sheep urine deposition is dependent on both the concentration of synthetic sheep urine applied and the grass species. The rhizospheric microbial community associated with plant roots is highly diverse, and it is conceivable that the complex plant-associated microbial community is important for plant health (Kyselková et al., 2009; Berendsen et al., 2012). The rhizospheric microbial community may contribute to maintaining plant health directly by releasing pathogen inhibitors, or indirectly by promoting plant growth (Kyselková et al., 2009; Berendsen et al., 2012). More specifically, the phylum *Proteobacteria* is involved in cycling of essential mineral nutrients (Lesaulnier et al., 2008; Chaudhry et al., 2012). For example, Chaudhry et al. (2012) showed that the higher abundance of *Proteobacteria* may contribute to improved soil fertility and plant growth. It has been reported that the phylum *Verrucomicrobia* acts as inhabitants of paddy soil (Asakawa and Kimura, 2008; Do Thi et al., 2012). However, little is known about the function of *Verrucomicrobia* in the semi-arid grasslands. Although its specific physiological functions in the soil remain to be clarified, the wide occurrence of this predominant phylum *Acidobacteria* across all samples may indicate a key role in soil ecosystem functioning. *Firmicutes* is related to plant health and the high abundance of *Firmicutes* can threaten plant fitness (Berendsen et al., 2012; Zhang et al., 2014). These results may indicate that these phyla in rhizosphere soil may be associated with the shift of plant community structure from co-dominance by a grass, *S. krylovii*, and a forb species, *A. frigida*, to exclusive dominance by the grass.

The arbuscular mycorrhiza (AM) is the mutualistic symbiosis between terrestrial plants and fungi. Fungi may play an important role in driving changes in plant biomass. Goomaral et al. (2013) reported that that *S. krylovii* increased in biomass as soil N availability increased, and this was associated with increased mycorrhizal colonization. Furthermore, altered fungal communities in response to environmental stressors in other systems have been linked to changing plant communities (Deslippe et al., 2012; Semenova et al., 2015). Therefore, further studies to investigate the involvement of fungi diversity and composition in N-induced shifts in species composition in the temperate steppe are warranted.

In the present study, to simulate the effects of N deposition on temperate grassland ecosystems, long-term N addition experiment was conducted in Inner Mongolia steppes by application of urea. The applied urea is first hydrolyzed to ammonia/ammonium by the enzyme urease in soils, and ammonium is further converted into nitrate by ammonia oxidizing bacteria and ammonia oxidizing archaea, leading to increases in inorganic N in soils (Zhang et al., 2012). Previous studies showed that application of urea in the Inner Mongolia steppes led to significant increases in soil nitrate concentrations (Fang et al., 2012). In addition, a similar reduction in plant species richness by addition of inorganic NH_4_NO_3_ in the Inner Mongolia steppes has been observed (Lan and Bai, 2012; Yang et al., 2012). Therefore, our N addition experiments with urea can simulate the natural N deposition.

In summary, our results demonstrated that long-term N addition had a profound influence on productivity of the steppe and composition of the rhizospheric microbial community of *S. krylovii* and *A. frigida*. These results highlight the importance of rhizospheric microbial communities in control and/or feedback of N addition-invoked steppe communities in response to N deposition. Our findings would contribute to our mechanistic understanding of impacts of N deposition on grassland ecosystem by linking changes in plant traits to their rhizospheric microbes-mediated processes.

### Conflict of interest statement

The authors declare that the research was conducted in the absence of any commercial or financial relationships that could be construed as a potential conflict of interest.

## Appendix

**Table A1:** **The number of reads before and after quality filtering**.

**Sample**		**Before quality filtering**	**After quality filtering**
N0 *S. krylovii*	1	151330	147221
	2	62531	60743
N2 *S. krylovii*	1	37876	36842
	2	88768	86471
	3	71252	69278
N8 *S. krylovii*	1	68660	66952
	2	51083	49787
	3	122252	118084
N16 *S. krylovii*	1	100960	97848
	2	118645	115232
	3	54686	53313
N0 *A. frigida*	1	83187	80841
	2	159845	155362
	3	80904	78636
N2 *A. frigida*	1	102194	99208
	2	77534	75623
	3	80417	78241
N8 *A. frigida*	1	72041	70274
	2	72393	70461
	3	72187	70604
N16 *A. frigida*	1	91416	89042
	2	81153	79019
	3	84889	82649
